# EvDTree: structure-dependent substitution profiles based on decision tree classification of 3D environments

**DOI:** 10.1186/1471-2105-6-4

**Published:** 2005-01-10

**Authors:** Jean-Christophe Gelly, Laurent Chiche, Jérôme Gracy

**Affiliations:** 1 Centre de Biochimie Structurale, Faculté de Pharmacie, Université Montpellier I, 15 avenue Charles Flahault, 34093 Montpellier Cedex 5, France

## Abstract

**Background:**

Structure-dependent substitution matrices increase the accuracy of sequence alignments when the 3D structure of one sequence is known, and are successful e.g. in fold recognition. We propose a new automated method, EvDTree, based on a decision tree algorithm, for automatic derivation of amino acid substitution probabilities from a set of sequence-structure alignments. The main advantage over other approaches is an unbiased automatic selection of the most informative structural descriptors and associated values or thresholds. This feature allows automatic derivation of structure-dependent substitution scores for any specific set of structures, without the need to empirically determine best descriptors and parameters.

**Results:**

Decision trees for residue substitutions were constructed for each residue type from sequence-structure alignments extracted from the HOMSTRAD database. For each tree cluster, environment-dependent substitution profiles were derived. The resulting structure-dependent substitution scores were assessed using a criterion based on the mean ranking of observed substitution among all possible substitutions and in sequence-structure alignments. The automatically built EvDTree substitution scores provide significantly better results than conventional matrices and similar or slightly better results than other structure-dependent matrices. EvDTree has been applied to small disulfide-rich proteins as a test case to automatically derive specific substitutions scores providing better results than non-specific substitution scores. Analyses of the decision tree classifications provide useful information on the relative importance of different structural descriptors.

**Conclusions:**

We propose a fully automatic method for the classification of structural environments and inference of structure-dependent substitution profiles. We show that this approach is more accurate than existing methods for various applications. The easy adaptation of EvDTree to any specific data set opens the way for class-specific structure-dependent substitution scores which can be used in threading-based remote homology searches.

## Background

With the sequencing of entire genomes and the exponential growth of sequence databases on the one hand, and the significant number of known folds compared to the putative number of possible folds in the fold space on the other hand, sequence-structure comparison is currently one main challenge of the post-genomic era. To this goal, 3D-environments were used by Eisenberg and coll. in the early 90s to build statistical potentials indicating the probability of finding each amino acid in a given structural environment as described by the secondary structure, the solvent accessibility and the polarity of neighboring atoms [1]. Such statistical potentials were successfully applied to protein fold recognition [1-4] or protein model evaluation [5,6], and were shown to improve the quality of sequence-structure alignments [7].

Statistical potentials describing the propensity of a residue pair to be at a given spatial distance have proved successful as well [8-14], but are more difficult to use as information to guide sequence-structure alignments using dynamic programming. On the contrary, residue preferences for position-dependent structural environments are easily implemented in alignment programs [7,15]. Recent improvements in this field were achieved by (i) optimizing definition and classification of the 3D-environments, and (ii) by constructing substitution matrices instead of residue preferences, i.e. taking into account the native residue type [15-17]. Indeed, it has been shown that amino acid substitutions are constrained by the structural environment, each environment displaying a distinct substitution pattern [18,19]. The use of 64 distinct substitution matrices corresponding to different 3D environments based on secondary structure, solvent accessibility and hydrogen bonding, combined with structure-dependent gap penalty and with global or local alignment algorithms, provides good performance to the FUGUE software in fold recognition approaches [15]. In this paper we investigate the use of decision tree algorithms to automate and improve the classification of structural environments. The automation will allow easy adaptation to any particular selected data set, opening a way for the construction of various specific substitution matrices. Indeed, it appears that one problem in the use of statistical potentials for structure prediction is their lack of universality [13]. It may thus be worthwhile to derive potentials specific to prediction problems or to protein classes. The automated derivation proposed here will facilitate such developments.

In the first part of the work we focus on automatically building and evaluating structure-dependent substitution scores. The emphasis is given to the development of a method for automatic selection of the most informative classifications of 3D environments in order to set up a versatile method allowing easy compilation of structure-dependent substitution scores for any given set of proteins. In a second part, the method is applied to a specific protein class, the small disulfide-rich proteins.

Decision trees have attracted our attention for several reasons. Knowledge acquisition and description language are optimized automatically and do not require human expertise during the learning process. Thanks to the hierarchical organization of the inferred trees, classifications are obtained quickly and the chain of decisions leading to the prediction is explicit and can be easily interpreted (in contrast to artificial neural networks for example). Decision tree learning algorithms are also robust since they partition the data recursively. The first dichotomies near the tree root are then based on many examples and are therefore statistically reliable. To handle noise and uncertainty, the trees can be pruned during a post-processing step to remove possible misleading clusters at the bottom of the tree. The research field on this topic is well-established and the number of applications of decision trees to real world is huge.

## Methods

### Structure-dependent substitution profiles

**Figure 1:**
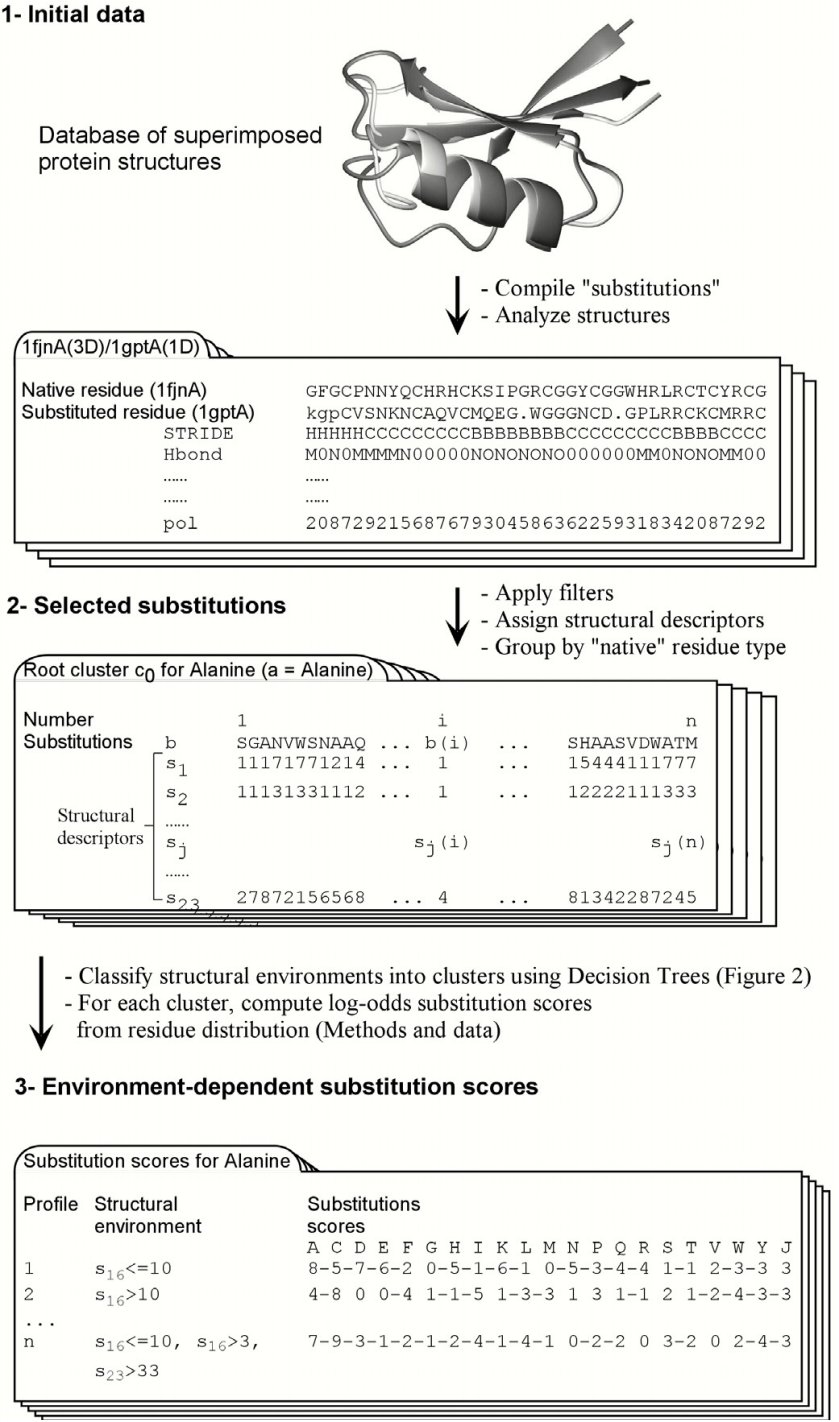
**Flow-chart of the EvDTree method. **1- Initial data are pairs of superimposed protein structures. For each position, the "native" residue in the reference protein, the "substituted" residue in the superimposed protein and structural parameters are tabulated. 2- The data are filtered and grouped according to the "native" residue type, resulting in root clusters for classifications. 3- For each residue type, a hierarchical classification is achieved using a decision tree algorithm. Substitution scores are computed for all resulting clusters from residue distribution in the cluster.

Standard substitution matrices are deduced from multiple sequence alignments of similar sequences [20-22]. To derive structure-dependent substitution matrices, multiple sequence alignments are also needed as well as a description of the 3D structure at each position in the alignment [15,17]. A schematic overview of the EvDTree method is displayed in Figure 1. Since we want to make sure that all residues at the same position in the alignment do share similar 3D structures, we will only use multiple alignments obtained from structural superimpositions (step 1 in Figure 1). From this, we extract all observed "substitutions" for each residue type and the corresponding structural environment (step 2 in Figure 1). The word "substitution" here is used to describe the residue replacement observed at an equivalent position in two structurally similar proteins. Then, for each residue type, the structural environments and associated substitutions are classified using a decision tree algorithm and substitution scores are computed from residue distributions observed in each cluster of the classification tree (step 3 in Figure 1).

**Figure 2:**
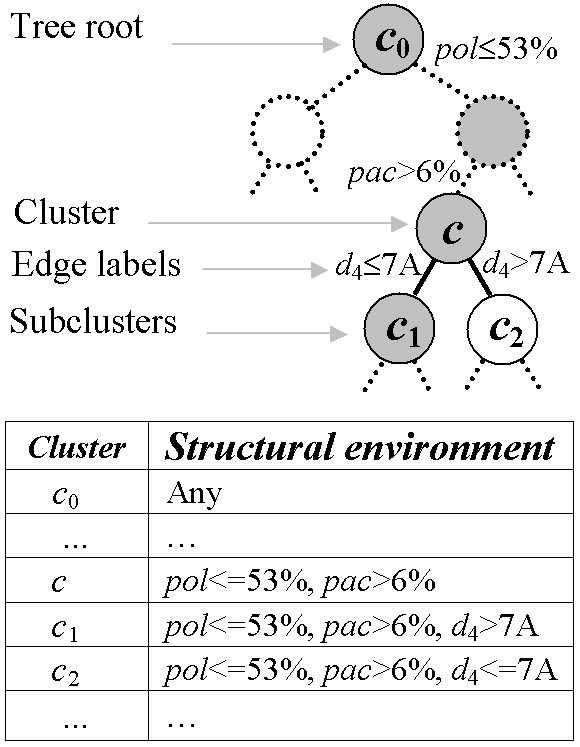
**Decision tree classification of all observed substitutions for Leucines. **Similar trees are built for each of the 21 residue types, including half-cystine. The structural environment for a given cluster is defined by the edge labels along its path from the root cluster *c*_0_. For example, the nodes colored in gray indicate the partial classification path of a Leucine observed in a native structural environment whose descriptors *s*_23_, *s*_15 _and *s*_10 _verify *s*_23 _= *pol *= 3%, *s*_15 _= *pac *= 7% and *s*_10 _= *d*_4 _= 5.4A.

Standard structure-dependent substitution matrices report the probability of 20 × 20 or 21 × 21 possible substitutions in a given structural environment [15]. In this work we classify 3D-environments and associated substitutions derived from alignments separately for each of the 21 residues (the 20 standard residues plus the half-cystine) using a decision tree algorithm (Figure 1, step 3 and Figure 2). As a result, we get several structure-dependent substitution profiles for each type of residue, that each indicates the relative probabilities of all 21 possible substitutions of one residue type in a given structural environment. Since the selected structural environments differ between residue types, the substitution profiles cannot be gathered into structure-dependent substitution matrices. As an example of how structural environments may differ between residues, a solvent-exposed environment might refer to a solvent accessibility > 6% if the residue is a leucine, but to a solvent accessibility > 33% if the residue is a glutamine.

### Learning and test data sets

Several data sets of structure-structure superimpositions and the corresponding alignments are available [23-25]. We have selected the database of homologous structure alignments, HOMSTRAD [23] for constructing both the learning and the test data sets of sequence-structure alignments. Main HOMSTRAD strengths are (i) a selection of accurate protein structures, (ii) a combination of automatic procedures and of manual inspections that guarantee low data noise, and (iii) its successful use in the derivation of the structure-dependent substitution matrices used in FUGUE [15]. Moreover, to facilitate comparison between our method and FUGUE, we selected the very same learning set previously used by Mizuguchi et al. This subset consists of 177 families extracted from the HOMSTRAD database and does not contain membrane proteins. From this HOMSTRAD subset, a set of 2.5 million of observed substitutions were extracted, one substitution corresponding to a residue in a reference structure, and the corresponding residue observed at the same position in a structurally similar protein. Moreover, to remove non-structural constraints from the sequence-structure alignments, the following filters were applied:

- Residues involved in a domain-domain or chain/chain interface, were excluded. Residues are considered to be involved in an interface when their solvent accessibility varies by more than 7 % when comparing the protein environment in the complex and in the isolated chain/domain. The cut-off value was taken from Mizuguchi et al [15], who used a similar filter to remove residues at a chain/chain interface.

- Residues that are not correctly superimposed in the structural superimposition were also excluded. The superimposition was considered good enough when the deviation between the two corresponding alpha carbons is below 3.5 Å. We assume that larger deviations may correspond to incorrect structural superimposition for the particular residue even though other residues are correctly aligned. Large deviations may also imply significant modifications in the 3D-environment. Although this 3.5 Å criterion is sometimes too restrictive, it actually leaves enough data for robust statistical estimations while removing most of aligned amino acid pairs whose respective structural contexts are not superposable.

Application of the two above filters excluded about 20% of the initial substitutions leaving about 2 million substitutions for the learning process. This data set was split into (i) a learning data set containing 950 000 substitutions similar to the learning set used by Mizuguchi et al. (ii) a pruning data set containing 325 000 substitutions, and (iii) a test data set containing 355 000 substitutions. The learning data set has been in some cases filtered further based on the percentage of sequence identity between superimposed proteins, resulting in smaller sets of 500 000 (0–40% id) or 700 000 (0–60% id) substitutions, respectively.

- Since we only work with three-dimensional structures, the oxidation state of any cysteine (free or disulfide bridged) is known. The symbol 'C' refers to disulfide bridged cysteines (half-cystines), whereas the symbol 'J' was used for free cysteines.

### Structural descriptors

Since the decision tree algorithm is able to automatically select the most discriminating structural descriptor at each classification step (see below) we do not need to empirically determine the 'best' descriptors. In this work, twenty-three structural descriptors were provided to the classification algorithm. The secondary structure (ss1) was assigned to each residue according to STRIDE [26] into seven categories. Values are as follows: ss1 = 1, 2, 3, 4, 5, 6 and 7 for α-helices (H), 3_10 _helices (G), π-helices (I), isolated bridges (B), extended conformations (E), turns (T) and coils (C), respectively. We also used a simpler 3-state description (ss2) deduced from the STRIDE assignment: ss2 = 1, 2 or 3 for helices (H or G), sheets (B or E) and coils (I, T, or C), respectively.

Hydrogen bonds were determined using the Hbond software [27]. Four different descriptors were used for different type of interactions: side-chain...main-chain O atom (hb1), side-chain...main-chain N atom (hb2), side-chain acceptor...side-chain donor (hb3) and side-chain donor...side-chain acceptor (hb4). For each interaction type, the number of interactions was used as the descriptor value. Here again a simpler description (lh) was also implemented that takes value of 0, 1, 2, or 3 if the side-chain of the residue makes no hydrogen bond, makes hydrogen bond(s) with side-chain atom(s), makes hydrogen bond(s) with main-chain atom(s) or makes hydrogen bonds with both side-chain and main-chain atoms, respectively.

Other structural parameters were obtained using the local program compilPDB [J.G.]. Beside the secondary structure, the local structure was also described by the Phi and Psi dihedral angles, and by Cα-Cα distances: d3 = Cα_i _- Cα_i+3_, d4 = Cα_i _- Cα_i+4_, d5 = Cα_i _- Cα_i+5_, d6 = Cα_i _- Cα_i+6_, d7 = Cα_i _- Cα_i+7_. Other descriptors were the buried surface area (bur), percent of accessibility (pac), contact area with carbon atoms (C), nitrogen atoms (N), oxygen atoms (O), sulfur atoms (S), positively charged atoms (pp), negatively charged atoms (nn), or polar atoms (pol).

For simplicity, these structural descriptors will now be called *s*_1 _to *s*_23_.

It should be noted that some structural descriptors are correlated (e.g., the Phi and Psi dihedral angles versus the d3 and d4 alpha carbon distances). However, this descriptive redundancy is not a problem since it is eliminated during the tree construction where the most informative descriptors only are selected, as explained below.

### Automated classification of structural environments using a decision tree algorithm

The native structural environments observed in the learning data set were classified for each of the twenty amino acids, plus the half-cystine, resulting in twenty-one independent decision trees (Figures 1 and 2). The use of these decision trees is as follows: let (*a*(*k*), ***s***(k)) the position *k *in a protein for which we want to score substitutions, *a*(*k*) the residue type and ***s***(*k*) = (*s*_1_(*k*),...,*s*_23_(*k*)) the structural environment description at this position. After the learning phase explained below, each tree node will be associated to particular structural descriptor *s*_*j *_and threshold *S *and will be linked by edges to two subnodes whose structural environments will be constrained respectively by the tests *s*_*j*_≤*S *and *s*_*j*_>*S*. The classification of (*a*(*k*), ***s***(k)) will be obtained by selecting the decision tree corresponding to residue type *a*(*k*) and then by running through the tree from its root node to an appropriate leaf following at each node the edge whose test, *s*_*j*_≤*S *or *s*_*j*_>*S*, is compatible with the value of the corresponding structural descriptor *s*_*j*_(*k*) (Figure 2). Contextual substitutions scores associated to the selected tree leaf, as explained in a further paragraph, will then evaluate each possible substitution of the amino acid *a*(*k*).

According to the standard data mining terminology, the predictive variables are therefore the native amino acid type and its associated structural descriptors and the dependent variable to be predicted is the substituted residue at this position. During the learning phase which we will now describe, the goal of the decision tree construction is to optimize the predictive power of the structural descriptor test chosen at each node and therefore to maximize the bias of the statistical distributions of the substituted residues associated to each subnode towards a few types of amino acids. Ideally, tree leaves should be associated to only one type of substituted amino acid, but this never happens in practice because of the tree depth limitation and the data set noise.

Let (*a*(*i*), ***s***(*i*), *b*(*i*)) be the *i*-th example of the whole learning data set where *a*(*i*) is a native residue, ***s***(*i*) = (*s*_1_(*i*),...,*s*_23_(*i*)) is its structural environment description and *b*(*i*) is the substituted residue as observed in a structurally similar protein at the same position as *a*(*i*). The main steps of the decision tree construction from the learning data set are as follows (Figure 2):

1. The decision tree for a given residue type *A *is initiated to a unique root node with an associated cluster *c*_0 _= {*i */ *a*(*i*) = *A *} grouping all examples with native residue type *A*.

2. For each tree cluster *c *do :

a. Test in turn each descriptor *s*_*j *_and each associated threshold *S *that creates possible dichotomies of *c *into two subclusters *c*_1 _and *c*_2_. If *s*_*j *_has continuous values, 9 possible thresholds *S *are chosen to create dichotomies *c*_1 _= {*i*∈*c*/*s*_*j*_(*i*)≤*S*} and *c*_2 _= {*i*∈*c*/*s*_*j*_(*i*)>*S*} corresponding to the 10^th^, 20^th^, ..., and 90^th ^percentiles of the statistical distribution of the considered descriptor. If *s*_*j *_is restricted to a few discrete categories, all possible dichotomies *c*_1 _= {*i*∈*c*/*s*_*j*_(*i*)==*S*} and *c*_2 _= {*i*∈*c*/ *s*_*j*_(*i*)! = *S*} are created, where *S *is one of each possible value of *s*_*j*_.

b. Select the optimal dichotomy from previous step which satisfies the tree constraints (see section (i) below) and minimizes the chosen splitting criterion (see section (ii) below).

c. Insert the new clusters *c*_1 _and *c*_2 _as nodes in the tree by linking them to cluster *c *with respective edges labeled {*s*_*j*_(*i*)≤*S*} and {*s*_*j*_(*i*)>*S*} or {*s*_*j*_(*i*) == *S*} and {*s*_*j*_(*i*)! = *S*}. The structural environment associated to a particular cluster will be defined by all edge labels from the tree root to the considered tree node or leaf (see figure 2).

3. Finally, prune the tree according to the selected pruning method and pruning data set (see section (iii) below).

It should be noted the choice of the optimal descriptor at a given tree level will depend on both the amino acid identity of the native residue and each structural descriptor previously chosen as splitting criteria along the tree path that leads to the considered node.

Main parameters in the classification are (i) the tree constraints, (ii) the splitting criterion, and (iii) the tree pruning method.

#### (i) Tree constraints

- Tree depth: as the learning process goes deeper in the tree, more and more specific clusters are created. Beyond a certain depth, the chance that the corresponding rules can be applied to new examples outside the learning set drops significantly, resulting in an overfitting of the available data since deep clusters won't have enough associated examples to derive statistically significant distributions. Therefore, to avoid wasting time to partition the data into smaller and smaller clusters, maximum tree depths of 2 to 6 were tested.

- Cluster cardinal: For the same reason as above, a minimum cardinal of examples was required for each cluster. We tested values between 200 and 1200 with increments of 200.

- Tree balancing: A restriction on uneven distributions of samples among two clusters from the same parent was applied to prevent the creation of unbalanced trees which would require higher depth to fully partition the data. This restriction is achieved by the parameter *sim*_*cc *_measuring the cluster cardinal similarity between two subclusters obtained by splitting :


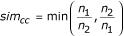


where, *n*_1 _is the cardinal of the subcluster 1 and *n*_2 _the cardinal of the subcluster 2.

#### (ii) Three different splitting criteria were tested

- The Gini criterion evaluates the probability that two randomly selected elements in a cluster correspond to two different types of residues [28]:


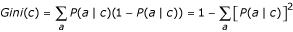


where *P*(*a*|*c*) is the relative frequency of residue type *a *in cluster *c*.

To evaluate the quality of a given segmentation into several clusters, the splitting criterion is given by


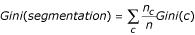


where *n*_*c *_is the number of elements in the cluster *c *and *n *is the total number of elements in all clusters.

- The Shannon entropy [29] tries to limit the distribution of elements of the same class among several clusters.


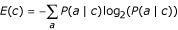


where *P*(*a*|*c*) is the relative frequency of residue type *a *in cluster *c*.

- We also used a specifically developed splitting criterion called the "mean rank" *MR*. Each class (residue type) in a cluster is ranked according to the number of elements of this class in the cluster (rank 1 is assigned to the most frequent residue type and rank 21 to the least frequent one). The mean rank *MR *evaluates the mean rank for a randomly selected element in the cluster. Low *MR *indicates clusters with only few well represented classes. Such clusters would correspond to structural environments that induce significant bias in the sequence and therefore strong structural constraints.


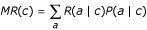


where *R*(*a*|*c*) and *P*(*a*|*c*) are the frequency rank and probability of the residue type *a *in the cluster *c*.

#### (iii) Three different pruning methods were considered

- The Pessimistic Error Pruning (PEP) [30] consists in recursively checking each cluster starting from the tree root and in cutting its corresponding subtree if this removal reduces the mean error rate estimated on the independent pruning test set by :


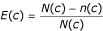


where *N*(*c*) is the number of examples assigned to the cluster *c *and *n*(*c*) is the number of occurrences of the most frequent amino acid in the cluster *c*. Let *C *be the father cluster from which *c *is derived in the tree, then *c *and its subtree will be removed if E(*c*)≥E(*C*).

- The Mean Rank Pruning (MRP) has a principle similar to PEP, except that *c *will be removed if *R*(*c*)<*R*(*C*) where *R*(*c*) and *R*(*C*) are respectively the mean ranks of the current cluster *c *and of its father cluster *C *averaged over the pruning test set.

- The pessimistic Mean Rank Pruning (PRP) is a more stringent version of MRP using a confidence margin to prevent statistically biased clusters to be kept in the tree. The current cluster *c *will now be removed if *R*(*c*)+ σ *t*_80_<*R*(*C*), where σ is the mean rank standard deviation over the pruning test set and the scaling factor *t*_80 _= 1.82 corresponds to a 80% confidence level for a Gaussian distribution.

#### Few other parameters were further optimized including

- A mutation weight α = 1/*N*_*f *_inversely proportional to the total number of residues *N*_*f *_in each protein family *f *of the learning data set. This insures that all structural families have similar importance in the derivation of the substitution probabilities.

- A mutation weight β = 25/*ide *inversely proportional to the percentage of identity *ide *between the two considered proteins. If *ide*<25%, then the mutation weight is decreased to 1. This reduces the importance of substitutions observed in similar sequences and could be used later to specialize EvDTree on different kinds of applications involving different sequence similarities.

### Residue specific environment-dependent substitution profiles

Once trees have been constructed, statistical distributions of observed substitutions in each cluster are used to compute cluster-specific environment-dependent substitution profiles. As explained previously, the structural environment associated to a particular cluster will be defined by all edge labels from the tree root to the considered tree node or leaf.

The probability for the amino acid *a *in the 3D environment *s *to be substituted by amino acid *b *is


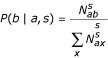


where 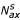 is the number of observed substitutions of amino acid *a *by amino acid *x *in the 3D environment *s*.

Smoothed probabilities *Q*(*b*|*a*,*s*) are then calculated as


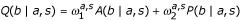


where *A*(*b*|*a, s*) is the *a priori *distribution of Topham et al. [19]. Relative weights are calculated as


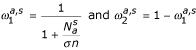


where 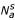 is the total number of occurrences of amino acid *a *in 3D environment *s*, *n *is the number of classes (21 in this case), and *σ *is a normalization constant. We used the value of 5 previously used by Topham et al. [19]. It should be noted that the weight of this "a priori" distribution is inversely proportional to the number of available examples 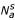 and is therefore maximum for undersampled substitutions.

Then the log odds scores are calculated as


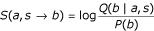


where *P*(*b*) is the background probability of occurrence of amino acid *b *in the whole database.

These log-odds are calculated for each node cluster of each native amino acid tree.

### Application and evaluation of environment-dependent and standard substitution scoring functions

To evaluate the EvDTree scoring function, each example of associated native residue, structural environment, substituted residue (*a*(*k*), ***s***(*k*), *b*(*k*)) from the test data set is classified by the tree corresponding to residue type *a*(*k*). Then the tree leaf corresponding to the structural environment *s*(*k*) is searched and its associated log-odds substitution scores are finally used to score the substituted residue *b*(*k*).

To compare the EvDTree substitution scores with other scoring methods, we have used the mean rank (*MR*) as the criterion to evaluate the quality of scoring functions. For each example in the test data set, the 20 possible substitutions are scored as indicated above, and the observed (real) substitution *b*(*k*) is ranked according to its score among all other possible substitutions. The mean rank over all examples in the test data set is indicative of how well the scoring function is able to recognize as probable the "real" substitutions. A MR of 1 would mean that the scoring function always gave the better score to the observed substitution. At the opposite, a MR of 10.5 would indicate that observed substitutions are scored randomly. The main advantage of the mean rank criterion is that it is fast to calculate and it is independent from the absolute values of the scores, therefore allowing comparisons between very different scoring functions. Similar criteria based on ranking were previously used to evaluate 1D-3D scoring functions [31].

The evaluation of environment-dependent substitution matrices requires computing the 3D environments the very same way they were computed when deriving the scores. Thanks to Dr K. Mizuguchi who provided us with all the necessary tools, we could include the FUGUE environment-dependent substitution matrices into our evaluation process.

To complement the *MR *evaluation, we also compared the performance of EvDTree with other scoring functions in sequence-structure alignments. To do this, 1000 sequence-structure alignments were selected from our test data set derived from the HOMSTRAD database. Each alignment was recalculated using a Smith and Watermann algorithm with several different substitution scoring functions. For each scoring function, the percentage of correctly aligned positions, according to the real alignments in the test data set, was compiled and used for comparisons. For each method, the gap opening (*Go*) and gap extension (*Ge*) penalties were optimized by comparing the alignments for several penalty combinations (*Go *= 2, 5, 10, 15, 20; *Ge *= 2, 5, 10, 15, 20).

## Results and discussion

### Decision tree classifications

Several learning data sets were compiled by filtering out observed protein substitutions (Figure 1) with sequence identity between superimposed proteins above thresholds of 40%, 60% or 80%. For each learning set, several combinations of parameters were tested for the construction of the EvDTree classifications and the calculation of the resulting structure-dependent substitution scores (Figures 1 and 2). For each run, a set of 21 decision trees was built and the corresponding scoring function was evaluated on the test data set using the mean rank (*MR*) criterion as explained in Methods. Due to the amount of data, CPU time limitations did not allow systematic examination of all parameter combinations, and the best parameters were determined through a limited trial and error protocol.

The variation of the mean rank over sixteen different runs remained limited (6.74 <*MR *< 6.89) showing that the method is robust and is not critically affected by slight modifications of the parameters. The lowest *MR *(6.74) was obtained with the following protocol:

- Minimum cardinal of any cluster = 600;

- The minimal cluster cardinal similarity between two subclusters obtained by splitting of the parent cluster is

*sim*_*cc *_= 0.1.

- Segmentation criterion: mean rank

- The pruning method is MRP.

- Examples in the learning set are weighted according to the number of residues in the protein family: α = 1/*N*_*f*_

- No weighting is done in relation to the sequence identity (β = 25/*ide*)

- Maximal sequence identity between superimposed proteins in the learning set = 60%

The values of the obtained mean ranks could, at first sight, appear rather high. However, it is worth noting that only substitutions were ranked in the evaluations, i.e. protein positions occupied by the same amino acid in the two structurally superimposed proteins were not considered in the evaluation process. They are included, however, in the statistics during the calculations of the substitution scores.

**Figure 3:**
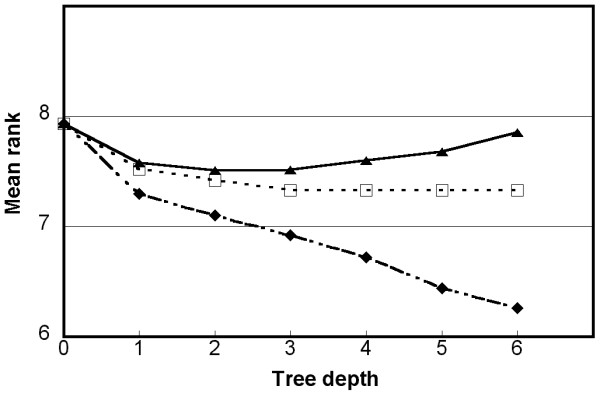
**Evolution of the Mean Rank criterion averaged over Alanine residues at different tree depths. **Values are indicated for the evaluations of the learning set (filled diamonds), of the test set before pruning (filled triangles) and after pruning the decision tree (open squares).

The evolution of the *MR *criterion along the learning process for alanine is shown in Figure 3. As expected, the MR decreases regularly when calculated on the learning set. On the other hand, when calculated on the test data set, the MR decreases in the first few learning steps, then increases in the following steps. The difference between the two curves in the last steps is due to overfitting, i.e. learning specific rules from the learning set that cannot be generalized, thus reducing the predictive power on the test data set. Pruning the tree using an independent pruning data set removes clusters with reduced predictive power resulting in a flat curve in the last steps.

### Analysis of the EvDTree classifications

For each residue type, the maximal tree depth used was 6, leading to a maximal number of leaves 21 × 64 = 1344, each of them potentially leading to a substitution profile that corresponds to one line in a classical substitution matrix. In other words, the total amount of data corresponds to 64 distinct substitution matrices, although it is not possible to associate substitution profiles to matrices since each profile corresponds to a different structural environment. The overall amount of data is nevertheless, in principle, comparable to the 64 environment-dependent substitution matrices used in the FUGUE system. However, the tree pruning step removed a significant number of clusters that do not afford improved information, leaving only 111 environments each associated to a specific substitution profile (data available from ).

Structural descriptors, thresholds and values used in the first dichotomies of the root clusters for each residue type are displayed in Table 1. Our main interest in using a decision tree classification is that, in principle, optimal splitting parameters and associated thresholds or values are automatically selected for each residue. As an example, it has been suggested that different boundaries on the fraction of area buried should be used for different residue classes when determining if a residue is exposed or buried [17]. Several examples of this can indeed be found in the EvDTree classifications: the selected structural descriptor for splitting the root cluster of serine and alanine substitutions is the percent of accessible area (pac), but the threshold is 3% for serine, whereas it is 10% for alanine (Table 1). The pac is also used as structural descriptor in the second dichotomy in the glutamic acid tree classification, with a threshold of 25%. The use of different accessibility thresholds by the decision tree algorithm fully supports previous observations by Rice and Eisenberg [17]. This observation also highlights the nice feature of decision trees that can be easily interpreted.

Analysis of the most discriminating structural descriptor selected in the learning process, i.e. the descriptor selected for the first dichotomy of the root cluster for each residue type (denoted c_0 _in Figure 2), shows that the secondary structure is the most discriminating parameter for aspartic and glutamic acids, lysine, arginine and asparagine (Table 1). Although contact polarity or solvent accessibility have been selected in subsequent dichotomies in most cases, it is clear that the substitution profile of charged residues primarily depends on the local structure. This result appears to be consistent with previous work by Gilis & Rooman [32] on the relative importance of local and non-local interactions in mutant stabilities. These authors showed that for solvent-exposed residues, the local structure is the most important factor, whereas distance potentials (i.e. 3D interactions) appear more suited to prediction of mutations in the protein core [32]. Here we show that substitution profiles for charged residues (which are largely solvent-exposed) mainly depend on the local structure. Another observation leads to a similar conclusion: for alanine and serine, the first selected structural parameter for splitting is the percent of accessibility (pac) and the most exposed resulting cluster is then split using secondary structure, whereas the most buried resulting cluster is split using the polarity of the protein environment (pol). These results confirm that, for solvent-exposed protein positions, the local structure is one main parameter that determines which amino acid can occupy this position.

**Figure 4:**
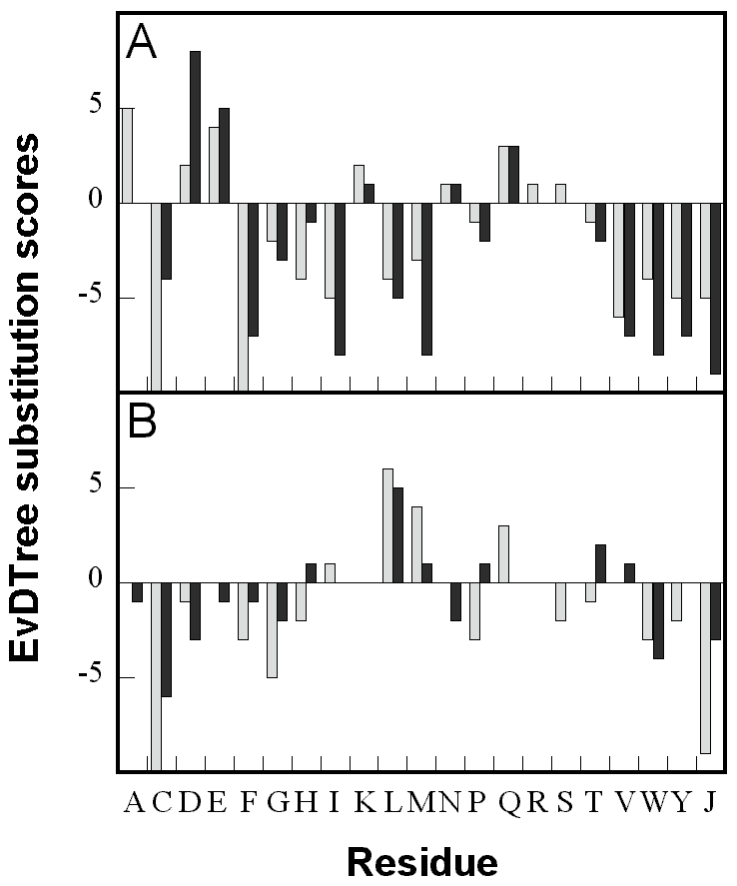
**Substitution profiles for 4 different EvDTree clusters. **(A) Two profiles for different amino acids (Ala and Asp) in similar structural environments corresponding approximately to an exposed α-helix. Grey bars: Ala with pac > 38 and ss1 = 1; Black bars: Asp with pac > 20 and ss2 = 1. (B) Two profiles for the same amino acid (Leu) in different structural environments. Grey bars: Leu with pol > 53 and ss1 = 1; Black bars: Leu with pol > 53 and ss1! = 1.

Four substitution profiles (i.e. log-odds substitution scores for one residue type into one structural environment) are displayed in Figure 4. Comparison of the substitution profiles for alanine and aspartic acid in similar environments (exposed α-helix) reveals significant differences (Figure 4A). This observation is not trivial since it could be postulated that, except for functional residues, the probability that a residue *b *occurs in a structural environment *s *only depends on *s *but is independent of the observed residue *a *in structurally similar proteins. The fact that, for similar structural environment, substitution profiles vary with the native residue probably indicates that purely structural descriptions probably lack some essential information, possibly related to the evolution process. This observation also illustrates the limits of environment-dependent statistical potentials in which the native amino acid is not taken into account. As an example, using data in Figure 4A, substitutions to Met in exposed α-helices appear more likely than substitutions to Leu when the native residue is Ala but the reverse is true when the native residue is Asp. Such differences cannot appear in environment-dependent statistical potentials such as 3D-1D scores that only describe the relative preference of residues for particular structural environments [1].

On the other hand, the substitution profiles for leucines in different structural contexts also display significant differences (Figure 4B). Thus, substitutions Leu → Met are favored in exposed α-helical positions whereas substitutions Leu → Thr are favored in exposed non α-helical positions (Figure 4B). This observation is not unexpected since it is well-known that β-substituted residues do not like to be in α-helices. Nevertheless it shows that EvDTree was able to extract consistent knowledge on sequence-structure relationships and it confirms previous observations that substitution scores are indeed structure-dependent [18,33], explaining why structure-dependent substitution matrices perform better than standard evolutionary matrices in fold recognition processes [1,15,17,34].

### Structural information improves prediction of substitution probabilities

The detailed impact of structural information for correct prediction of substitution probabilities can be approached by comparing the EvDTree substitution profiles with the evolutionary substitutions matrices GONNET [20] and BLOSUM62 [35]. To this goal, the "Mean Rank" criterion has been used (the lower the Mean Rank, the better the scoring function; see Methods and Data). Results by residue type and averaged over all residues are shown in Table 2. Comparison of EvDTree with the Gonnet and BLOSUM62 matrices shows that EvDTree performs clearly better on average, and individually for most residues. Therefore, the use of the structural information in EvDTree does improve the predictive power of substitution profiles versus structure-independent substitution matrices. Moreover, a substitution matrix was built from the tree clusters of EvDTree, i.e. before any structural information is taken into account. This matrix, referred to as EvDTree0, is simply derived from the structural superimpositions in the learning data set and is thus similar to other structure-derived substitution matrices [36]. Interestingly, EvDTree0 performs better than evolutionary matrices suggesting that, despite a lower amount of data, structure-derived alignments can provide data of higher quality than sequence alignments for derivation of substitution matrices.

The results in Table 2 show that EvDTree provides poorer evaluation than the evolutionary matrices for two residues, histidine and lysine, possibly due to an insufficient amount of data. It is also worth noting that histidine often participates in active sites or coordination sites, and the substitution probabilities may have been biased by this peculiarity. Filtering out the learning data set for coordination sites was performed by Shi et al [15], but such a filter was not implemented here.

More surprisingly, five residues (Gly, Met, Pro, Gln, and Thr) and the free cysteine (J) do not display evaluation improvement by using structural information (compare EvDTree0 and EvDTree). The latter remark means that for these five residues no structural descriptor permitted efficient splitting of the data. It is likely that for these residues new descriptors or descriptor combinations remain to be discovered. Nevertheless, on average, the structural information significantly improves the performance and EvDTree appears as a clearly better scoring function than evolutionary matrices in evaluation of sequence-structure alignments.

### The EvDTree substitution profiles provide slightly better substitutions predictions

Comparison with structure-dependent substitution matrices obtained by other groups is not as simple as for standard substitution matrices, because we must make sure that we compute the structural environment exactly the same way that was used for generating the matrices. We were able to compare EvDTree with FUGUE, thanks to the FUGUE accessory programs kindly provided by Dr K. Mizuguchi.

**Figure 5:**
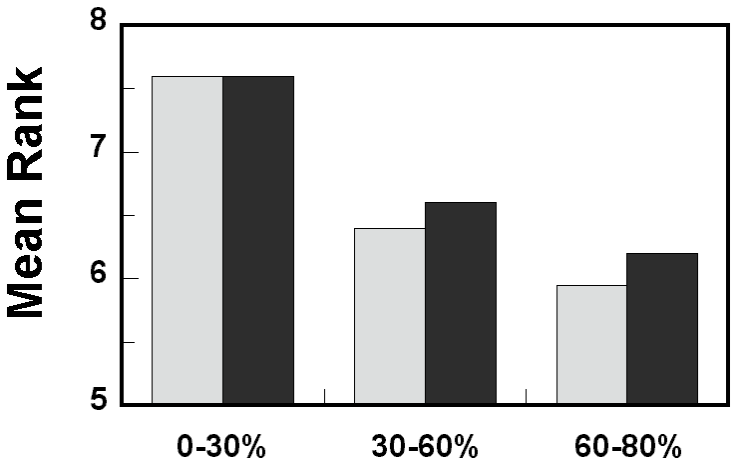
Comparison between EvDTree (grey) and FUGUE (black) at different levels of sequence identity in the test data set.

As shown in Table 2, the overall performance of EvDTree and FUGUE appear very similar. A comparison between these two scoring functions at different levels of sequence identity is displayed in Figure 5. EvDTree provides slightly better performances at sequence identity above 30% but similar results below 30% (Figure 5). The reason for this remains unclear, but might be a result of our filtering of the learning set that removed all positions were Cα deviates from more than 3.5 A. Further optimization of the EvDTree learning set would probably be necessary for use in fold recognition programs at low sequence identity. The observation that EvDTree performs at least as well as the scoring function of FUGUE but is computed in a fully automated manner opens the way for future potential applications. For example, we show below that EvDTree can easily optimize fold-specific scoring matrices specific of small disulfide rich proteins leading to improved substitution scores for this particular class of proteins.

### Evaluation of EvDTree as scoring function in sequence-structure alignment

**Figure 6:**
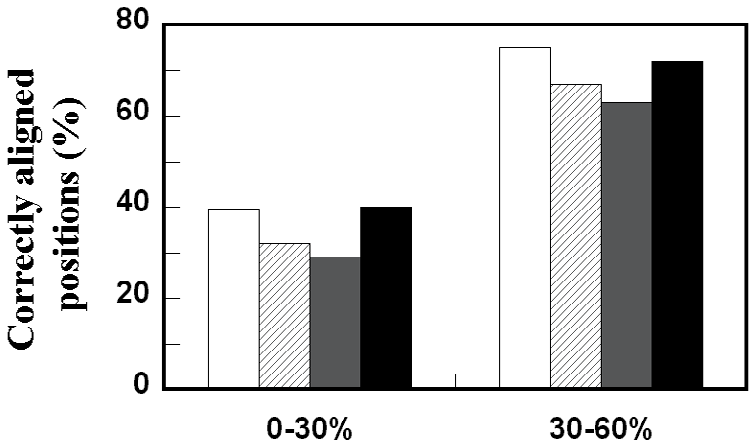
**Comparison of sequence-structure alignments using several scoring functions. **EvDTree, white bars; Gonnet, hatched bars; BLOSUM62, grey bars, and FUGUE, black bars. Gap opening and gap extension penalties have been separately optimized for each scoring function.

Although the Mean Rank test determines the ability of a scoring function to correctly evaluate structure-compatible sequences, it does not determine how well a scoring function would actually perform in a particular application, e.g. fold recognition. In this paper, we do not focus on a particular application, but rather on the method to automatically derivate structure-dependent substitution profiles, and the Mean Rank appears as a simple and efficient criterion to rapidly compare different learning parameterizations or scoring functions. To verify that the theoretical evaluations using the Mean Rank have some significance for future real applications, we compared EvDTree with other methods used as scoring functions in sequence-structure alignments. The percent of correctly aligned positions for different methods and for different sequence identity ranges is displayed in Figure 6. For each method, the best Gap opening and Gap extension penalties were roughly optimized by checking 25 combinations (*Go *= 2, 5, 10, 15, 20; *Ge *= 2, 5, 10, 15, 20). The results shown in Figure 6 fully confirm previous analyses using the Mean Rank criterion: on average, better alignments are obtained using the structure-dependent scoring functions FUGUE and EvDTree, and EvDTree provides slightly better alignments than FUGUE above 30% of sequence identity but similar results at lower sequence identity. The slightly better accuracy of EvDTree for mid-range percentages of identities suggests that our approach could be particularly useful to improve sequence/structure alignments in homology modeling. The EvDTree learning algorithm could also be applied to fold recognition by optimizing scoring functions specific of particular protein families.

### Application of EvDTree to a specific class of proteins, the small disulfide-rich proteins

The strongest potential of EvDTree is its ability to adapt itself to any particular set of structures. As a test case, we have applied EvDTree to small disulfide-rich proteins. Small disulfide-rich proteins display several peculiar structural features: (i) due to their small size, a larger number of residues than usual are solvent-exposed, (ii) regular secondary structures are limited and the content in turns and loops is high, (iii) the hydrophobic core is largely constituted by the disulfide bridges that are responsible for the high stability despite the small size, and (iv) glycine, proline, and, of course, cysteine residues are more frequent than usual. With all these peculiarities, small disulfide-rich proteins are not well-suited to standard prediction methods and it has been shown that these proteins score poorly using the standard PROCHECK database [37]. Evaluations of non-specific substitution scoring functions on small disulfide-rich proteins using the mean rank criterion are reported in Table 3. Comparison of values in Table 3 for EvDTree, Gonnet, BLOSUM62 and FUGUE with those in Table 2 clearly support the idea that small disulfide-rich score poorly when using standard databases and methods.

Thus, to test the ability of EvDTree to automatically adapt itself to a class of proteins with specific structural features, we have computed disulfide-rich specific substitution profiles (EvDTreeDS). For this, we have compiled a specific data set from the structural class "small disulfide" in the HOMSTRAD database. These data were complemented by data extracted from the KNOTTIN database [38]. The number of structural positions in the initial data set (about 40000) is clearly insufficient to divide this set into independent learning and test datasets. Therefore, a "leave-one-out" protocol was used. In this protocol, one protein family is excluded from the learning set and the resulting substitution scores are used to evaluate this family. This process is repeated for all protein families in the initial set. Also, due to the limited number of data, the tree pruning had to be performed using the learning data set, which is of course far less efficient than using an independent data set (Esposito, 1997), and resulted in a very limited pruning when compared to the general case.

Mean rank evaluations of the new, specific, EvDTreeDS substitution scores on small disulfide-rich proteins are reported in Table 3. Despite the limited set of data, comparison of the efficiency of the EvDTreeDS specific substitution profiles with standard EvDTree clearly shows that the automated classification method was able to extract, at least in part, the specific features of the small disulfide-rich proteins.

Furthermore, the comparison of the structural descriptors used in the first partition of root clusters in EvDTree and EvDTreeDS classifications highlights interesting differences (Table 1). First, when the solvent accessibility percentage is the first used descriptor in both trees (half-cystines and alanines), the thresholds retained by the learning algorithm are different for the disulfide-rich proteins which are small and whose residues, except half-cystines, are more exposed to solvent on average. Accordingly, the selected threshold is lower for half-cystines but higher for alanines.

Table 1 also reveals that most large hydrophobic residues (Leu, Phe, Tyr, His) switch their most discriminating descriptor from hydrophobic-hydrophilic measures in EvDTree (Pol, C) to secondary structure descriptors in EvDTreeDS (ss1 and ss2). This inversion can be interpreted by the reduction of the buried volume in the small disulfide-rich structures which cannot accommodate for large residues. This suggests that, in these proteins the stability is mainly due to disulfide bridges whereas the hydrophobic effect would be less crucial. Being more solvent exposed and less involved in hydrophobic packing, large hydrophobic residues might become more sensitive to local structure in small disulfide-rich proteins.

More surprisingly, we also notice that charged and polar residues (Asn, Asp, Glu, Lys, Arg) display the opposite switch, i.e. a secondary structure descriptor is used in EvDTree but a solvent accessibility descriptor is used in EvDTreeDS. Analysis of the EvDTreeDS classification suggests that these residues, when in more buried positions, are far more conserved than when in more exposed positions. We think that beside the disulfide bridge core, additional elements of stability often occur through specific hydrogen bonding networks between charged or polar residues and the backbone, rather than through hydrophobic packing. Typical examples of this are the conserved glutamic acid in position 3 of cyclotides [39], or the conserved aspartic acid in position 15 of squash inhibitors [40] which both participate in multiple hydrogen bonding with the backbone. This might explain, at least in part, why the distinction between exposed and partially buried charged residues is more critical in small disulfide-bridged proteins.

All these subtle modifications revealed by the EvDTree and EvDTreeDS classifications suggest that there is probably no universally optimal description language and that the choices and partitions of structural descriptors should be adapted to the class of proteins considered. Our new decision tree learning algorithm makes this fine tuning automatically from scratch whereas classical potentials are based on globally optimized description languages which may become suboptimal in specific contexts.

## Conclusions

We have described a new method, EvDTree, based on decision tree classification of structural environments to automatically construct structure-dependent substitution profiles from a set of sequence-structure alignments. The EvDTree method was shown to perform similarly to the successful environment-dependent substitution matrices used in FUGUE (Shi *et al.*, 2001). Interestingly, the tree-pruning step removed a significant number of structural clusters yielding an average tree depth of 4 instead of the six allowed levels. This is an indication that clusters at higher levels corresponded to the learning of specific sequence-structure relationships that could not be generalized (Figure 3). It may be expected that as more high quality data will become available, this effect could be reduced and higher levels of the decision tree will gain better performances.

In this work, we were interested in the development of a fully automatic method for the classification of structural environments and inference of structure-dependent substitution profiles. The evaluation of the intrinsic performance of the substitution profiles was primarily done on known sequence-structure alignments, using the mean rank of observed substitutions. We have shown that in this context, the EvDTree substitution profiles perform slightly better than other successful substitution matrices, and as such, the EvDTree matrices constitutes interesting elementary data for various applications. Moreover, comparison of the EvDTree substitution scores with other scoring functions for sequence-structure alignments led to results similar to the mean rank evaluation supporting the usefulness of the latter criterion.

One specific strength of the EvDTree method is its easy automatic adaptation to any specific data set. Here we have shown that it is possible to obtain structure-dependent substitution profiles specific of small disulfide-rich proteins with better predictive power than standard substitution scores. This approach could be easily extended to other specific protein classes such as coil-coils, membrane proteins, etc. as soon as enough structures are available for learning. Fold-specific substitution matrices have recently been proposed for protein classification [41]. The EvDTree approach opens the way for class-specific or fold-specific structure-dependent substitution scores for use in threading-based remote homology searches. Decision trees based on different learning sets and with different depths could be optimized depending on the available protein structures and sequences of the fold family considered.

The fact that, as stated above, the structural information did not yield better prediction for several residues in the EvDTree approach (Table 2) suggests that improvements are still possible. To this end, 3D environments from the decision trees yielding poor performances should be determined in order to design more appropriate structural descriptors. It is tempting to speculate that using combined structural descriptors, e.g. (Phi, Psi) angle pairs which can delineate particular regions of the Ramachandran plot or (dCi,i+j, dCi,i+k) distance pairs which can introduce some super-secondary structural constraints, could increase the accuracy of the decision trees. Alternatively, the use of linear combinations of descriptors in decision tree induction algorithms have been reported and could be used for structural classifications [42].

## Authors' contributions

After an initial program from JG, JCG coded a new version of the software to incorporate a novel algorithm. All methods were implemented and tested by JCG. The whole work was conceived by JCG, JG and LC and was supervised by LC and JG. All authors read and approved the final manuscript.

## Figures and Tables

**Table 1:** Structural descriptors and associated values or thresholds used by EvDTree to partition the root cluster *c*_0 _for each residue type into two subclusters (see Figure 2). The EvDTreeDS columns refer to the decision tree classification of a learning set containing only small disulfide-rich proteins (see text).

**Amino acid**^**a**^	**EvDTree**			**EvDTreeDS**		
	
	Structural descriptor	Value		Structural descriptor	Value	
				
		subcluster 1	subcluster 2		subcluster 1	subcluster 2
C^**b**^	pac	<= 8	> 8	pac	= 0	> 0
A^**b**^	pac	<= 10	> 10	pac	<= 35	> 35
I	pol	<= 41	> 41	C	<= 35	> 35
L^**c**^	pol	<= 53	> 53	ss1	= 6	≠ 6
F^**c**^	C	<= 65	> 65	ss2	= 2	≠ 2
Y^**c**^	C	<= 65	> 65	ss2	= 3	≠ 3
H^**c**^	C	<= 35	> 35	ss2	= 3	≠ 3
V	pol	<= 46	> 46			
M				ss1	= 4	≠ 4
W	bur	<= 155	> 155	N	<= 1	> 1
N^**d**^	ss2	= 1	≠ 1	pac	<= 49	> 49
D^**d**^	ss2	= 1	≠ 1	bur	<= 25	> 25
E^**d**^	ss1	= 4	≠ 4	pac	<= 54	> 54
K^**d**^	ss2	= 2	≠ 2	pac	<= 53	> 53
R^**d**^	ss1	= 1	≠ 1	bur	<= 101	> 101
S	pac	<= 3	> 3			
T				pac	<= 49	> 49
G				ss1	= 7	≠ 7
Q				ss2	= 3	≠ 3
P				C	<= 7	> 7

^a^Only dichotomies for which the structural information improves the Mean Rank evaluation are shown (Tables 2 and 3). ^b^Residues for which the same descriptor is used in EvDTree and EvDTreeDS. ^c^Residues for which a descriptor corresponding to the local structure has been used by the EvDTreeDS but not by the EvDTree learning process. ^d ^Residues for which a descriptor corresponding to the local structure has been used by the EvDTree but not by the EvDTreeDS learning process.

**Table 2:** Mean rank evaluation of the structure-dependent EvDTree substitution scores and comparison with other scoring functions.

**Amino acid**	**DT0^b^**	**DT^a^**	**GON^a^**	**B62^a^**	**Fug^a^**
**A**	7.93	**7.33**	8.21	8.34	7.51
**C**	10.62	10.60	11.01	11.86	**10.19**
**D**	6.48	**6.41**	6.85	6.59	6.48
**E**	6.83	**6.73**	6.92	6.95	6.79
**F**	6.66	**6.58**	7.04	6.86	7.01
**G**	7.14	7.14	7.16	7.20	**7.00**
**H**	8.18	8.05	8.29	7.98	**7.97**
**I**	5.65	**5.38**	5.88	5.72	5.58
**K**	6.97	6.93	6.88	6.86	**6.81**
**L**	6.71	**6.39**	6.58	6.69	6.52
**M**	**5.68**	**5.68**	6.01	5.84	5.87
**N**	6.82	**6.73**	7.20	7.03	6.90
**P**	**7.15**	**7.15**	7.58	7.80	7.33
**Q**	**6.74**	**6.74**	7.08	7.28	7.29
**R**	7.46	7.24	7.41	7.41	**7.21**
**S**	6.43	**6.32**	7.03	6.73	6.67
**T**	**6.61**	**6.61**	7.21	6.74	7.00
**V**	6.50	**6.28**	6.65	6.38	6.36
**W**	8.03	**7.61**	8.81	9.04	7.63
**Y**	8.38	8.11	9.15	8.82	**8.02**
**J**	**7.12**	**7.12**	8.17	7.48	7.16
**Average**	6.90	**6.74**	7.15	7.06	6.87

^a^Mean rank evaluation of the EvDTree (DT), Gonnet (Gon), Blosum62 (B62) and FUGUE (Fug) substitution scores. ^b^Mean rank evaluation of EvdTree0 (DT0), i.e. scores inferred from the root clusters before structural classifications. Bold numbers indicate the best values among the methods.

**Table 3:** Mean rank evaluation of small disulfide-rich proteins using standard and specific scoring functions.

**Amino acid**	**DT^a^**	**DS0^a^**	**DS^a^**	**Fug^a^**	**Gon^a^**	**B62^a^**
**A**	8.16	8.59	**7.71**	8.39	8.85	8.83
**C**	9.84	11.60	**8.09**	10.42	11.8	11.72
**D**	**6.85**	7.50	7.31	6.90	7.12	7.01
**E**	**6.70**	7.57	7.22	6.89	6.88	6.89
**F**	8.58	8.77	**7.58**	10.14	9.79	9.86
**G**	8.03	7.74	**7.35**	7.48	7.40	7.46
**H**	7.99	8.04	**7.51**	8.00	7.72	8.09
**I**	7.74	8.55	**7.28**	7.82	8.36	8.19
**K**	7.47	8.07	7.56	7.49	7.59	**7.36**
**L**	9.13	9.41	**8.34**	9.25	9.70	9.90
**M**	9.74	9.04	**7.33**	8.95	9.16	8.87
**N**	7.57	8.14	8.09	**7.411**	8.40	8.02
**P**	8.20	8.41	**8.00**	8.11	8.70	8.56
**Q**	**6.88**	7.94	7.81	7.35	7.38	7.38
**R**	7.93	8.20	**6.96**	7.84	7.99	8.11
**S**	7.56	**7.55**	**7.55**	7.56	8.29	8.17
**T**	8.14	8.61	**7.63**	8.53	9.40	8.91
**V**	8.30	**7.69**	**7.69**	7.82	8.76	8.48
**W**	8.78	7.46	**7.45**	8.20	9.03	8.82
**Y**	8.61	8.59	**7.53**	8.76	9.77	9.32
**Average**	7.90	8.27	**7.59**	8.02	8.44	8.38

^a^Mean rank evaluation using the EvDTree (DT), EvDTreeDS0 (DS0), EvDTreeDS (DS), FUGUE (Fug), Gonnet (Gon) and Blosum62 (B62) substitution scores. Bold numbers indicate the best values among the methods.
